# Association of domain-specific physical activity with depressive symptoms: A population-based study

**DOI:** 10.1192/j.eurpsy.2022.2350

**Published:** 2022-12-12

**Authors:** Fan He, Yi Li, Zixin Hu, Hui Zhang

**Affiliations:** 1 Human Phenome Institute, Zhangjiang Fudan International Innovation Centre, Fudan University, Shanghai, China; 2 Artificial Intelligence Innovation and Incubation Institute, Fudan University, Shanghai, China; 3 National Clinical Research Centre for Ageing and Medicine, Huashan Hospital, Fudan University, Shanghai, China

**Keywords:** Depressive symptoms, domain-specific physical activity, epidemiology

## Abstract

**Background:**

It remains unclear whether all physical activity (PA) domains (e.g., occupation-related PA [OPA], transportation-related PA [TPA], and leisure-time PA [LTPA]) have equivalent beneficial relationships. We aimed to investigate the associations of OPA, TPA, and LTPA with depressive symptoms in adults.

**Methods:**

We included and analyzed 31,221 participants (aged ≥18 years) from the cross-sectional 2007–2018 U.S. National Health and Nutrition Examination Survey (NHANES). The PA domains were assessed by a self-report questionnaire and categorized based on the PA guidelines. Depressive symptoms were measured by the nine-item Patient Health Questionnaire.

**Results:**

Participants achieving PA guidelines (≥150 min/week) were 26% (odds ratio [OR] 0.74, 95% confidence interval [CI] 0.68–0.80) and 43% (OR 0.57, 95% CI 0.51–0.62) less likely to have depressive symptoms depending on total PA and LTPA, respectively, while OPA or TPA did not demonstrate lower risks of depressive symptoms. LTPA at levels of 1–149, 150–299, and ≥300 min/week was associated with 31% (OR 0.69, 95% CI 0.60–0.78), 43% (OR 0.57, 95% CI 0.49–0.67), and 51% (OR 0.49, 95% CI 0.43–0.55) lower odds of depressive symptoms, respectively.

**Conclusion:**

LTPA, but not OPA or TPA, was associated with a lower risk of depressive symptoms at any amount, suggesting that significant mental health would benefit from increased PA, even at levels below the recommendation.

## Introduction

Depression is a common mental disorder that affects approximately 322 million people in the general population [1]. The Global Burden of Disease Study reported that depression is the leading cause of mental health-related disability burden worldwide [2]. Additionally, depressive symptoms are still on the rise and are associated with all-cause and cardiovascular disease mortality among adults [3–5]. Therefore, an effective intervention strategy is vital for the prevention or treatment of depressive symptoms [6, 7]. Emerging research has linked depressive symptoms to lifestyle factors, such as physical activity (PA), diet, smoking, and sleep [8–10]. Among these potentially beneficial lifestyles, PA was demonstrated to be a low-risk augmentation therapy for depressive symptoms [11]. For example, prospective cohort studies reported that increased PA is associated with reduced risks of depressive symptoms [12–14]. Mendelian randomization studies also demonstrated a potential protective role of PA on depressive symptoms [15, 16].

PA is a complex behavior that consists of different domains, including occupation-related PA (OPA), transportation-related PA (TPA), and leisure-time PA (LTPA). Different PA domains may contribute to health through different ways throughout life, such as diabetes [17, 18], nonalcoholic fatty liver disease [19, 20], significant fibrosis [20], and mortality [21–23]. However, studies on PA and depression have generally focused on total PA or LTPA, and few studies have investigated whether the same health-enhancing relationships are observed across different PA domains in the general population [12, 13]. For example, a recent meta-analysis of 111 prospective cohort studies reported that customary and increasing levels of LTPA are inversely associated with incident depression among adults [13]. In addition, Ryu et al. [24] reported that higher leisure and transport PA were related to lower levels of depressive symptoms, while higher work PA was associated with higher levels of depressive symptoms in the Korea National Health and Nutrition Examination Survey. However, Cocker et al. [25] found favorable associations between any domain (leisure‐time, transport‐, and work‐related) of PA and depressive symptom severity according to the European Health Interview Survey. In summary, it remains unclear and controversial whether all domains of PA (e.g., OPA, TPA, and LTPA) have equivalent beneficial relationships on depressive symptoms.

This study addresses this knowledge gap by investigating associations between different PA domains (including OPA, TPA, LTPA, and total PA) and the risk of depressive symptoms in adults across a wide age spectrum. We hypothesized that each domain of PA would have beneficial relationships with depressive symptoms and examined whether the association between the related domain of PA and depressive symptoms would differ by age, gender, body mass index (BMI), race, economic level, marital status, and smoking status. Additionally, we assessed the dose–response association between the related domain of PA and depressive symptoms.

## Methods

### Study population

**Figure 1.**
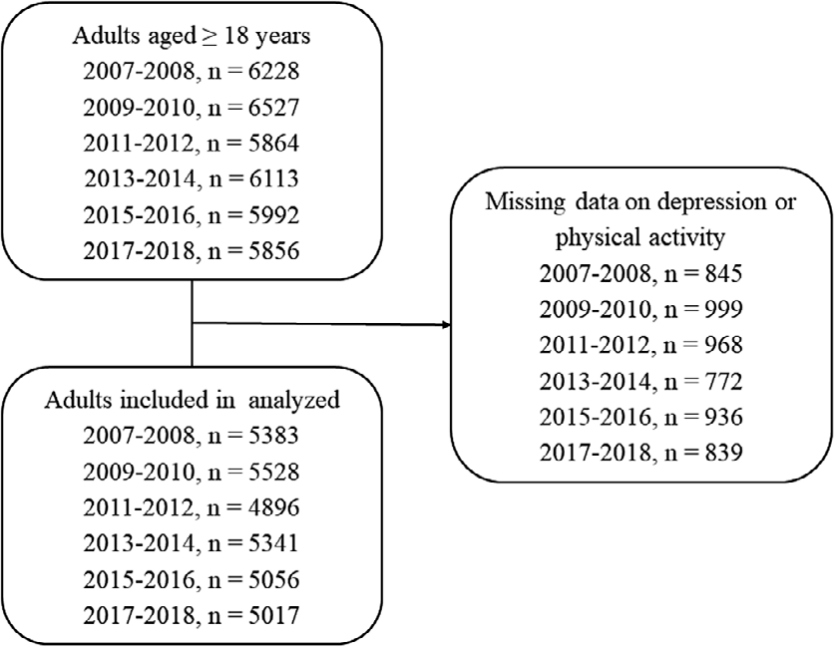
Flow diagram of the included survey participants.

The National Health and Nutrition Examination Survey (NHANES) is a nationally representative cross-sectional survey of civilian, noninstitutionalized persons living in the US [26]. We used data from the NHANES 2007–2008, 2009–2010, 2011–2012, 2013–2014, 2015–2016, and 2017–2018 (Figure 1). Participants aged 20 years or older with complete nine-item Patient Health Questionnaire (PHQ-9) and a self-reported questionnaire of PA were included. The final sample included 31,221 individuals. Because the data in our study were publicly available and performed as a secondary analysis, no additional ethical review was necessary.

### Physical activity

PA was self-reported by participants using the Global Physical Activity Questionnaire [27], which assessed three PA domains: OPA, TPA, and LTPA. The questionnaire about the frequency (time per week), duration (minutes per time), and intensity (vigorous or moderate) of PA in one typical week was measured for OPA and LTPA. Minutes of the vigorous intensity of PA were doubled and added to minutes of the moderate intensity for OPA and LTPA [18].

The total PA was defined as the sum of the OPA, TPA, and LTPA. PA was categorized according to the 2018 PA guidelines, which recommend that adults should engage in at least 150–300 min per week of moderate-intensity PA, 75–150 min per week of vigorous-intensity PA, or an equivalent combination [28]. In brief, the participants were dichotomized into two groups: (1) participants who met the 2018 PA guidelines (the summed minutes of PA were 150 min or more in a typical week) and (2) participants who did not meet the 2018 PA guidelines (the summed minutes of PA were less than 150 min in a typical week). To assess the dose–response association between related domains of PA and depression, we also categorized the summed minutes of PA into four groups: (1) 0 min/week, (2) 1–149 min/week, (3) 150–299 min/week, and (4) ≥300 min/week based on previous reports [18–20].

### Depression

Depressive symptoms were assessed by PHQ-9 [29, 30], which assesses frequencies at certain symptoms experienced in the last 2 weeks, ranging from 0 (“not at all”) to 3 (“nearly every day”). These questionnaires were self-administered, and the total scores ranged from 0 to 27. A cut-off score of 10 was used to define probable cases of depressive symptoms, with a sensitivity of 88% and a specificity of 88% [29].

### Covariates

Covariates included sociodemographic information, lifestyle behaviors, and clinical characteristics. In detail, sociodemographic information included age, gender (male versus female), marital status (married or living with partners versus single), education (less than college versus college or more), race/ethnicity (Mexican Hispanic, Other Hispanic, Non-Hispanic White, Non-Hispanic Black, and Other race), and poverty income ratio (<1, 1–1.99, 2–4, and ≥4). The poverty index ratio, calculated by dividing family income by poverty guidelines specific to family size, year, and state, was based on the Department of Health and Human Services’ poverty measure [26]. Lifestyles included smoking status (never, former, and current smokers) and BMI (low to normal, overweight, and obese). Clinical characteristics included hypertension, diabetes, heart disease, stroke, pulmonary disease, and cancer. Hypertension was defined as having at least one of the following: systolic blood pressure ≥140 mmHg, diastolic blood pressure ≥90 mmHg, and having been diagnosed by a doctor or other health professional. Diabetes was defined as being told by a doctor or health professional that they had diabetes, blood glycosylated hemoglobin (HbA1c) ≥ 6.5%, fasting plasma glucose ≥126 mg/dL, or random plasma glucose ≥200 mg/dL [31]. Arthritis, stroke, cancer, congestive heart failure, coronary heart disease, angina/angina pectoris, heart attack, asthma, emphysema, chronic bronchitis, and chronic obstructive pulmonary disease were defined as being told by a doctor or health professional that they have the disease. Heart disease included congestive heart failure, coronary heart disease, angina/angina pectoris, and heart attack, and pulmonary disease included asthma, emphysema, chronic bronchitis, and chronic obstructive pulmonary disease.

### Statistical analysis

Continuous and categorical variables are presented as the means with standard deviation (SD) or frequency (%), respectively. Group differences were analyzed by Student’s *t-*test or ANOVA. Logistic regression models were used to evaluate the association between PA and the risk of depression. All analyses were conducted using three age- and sex-adjusted models. Multivariable Model 1 was adjusted for age, sex, BMI, race, education level, marital status, smoking status, poverty ratio, and years of NHANES. Multivariable Model 2 was adjusted for stroke, diabetes, arthritis pulmonary disease, hypertension, heart disease, cancers, and polypharmacy in addition to multivariable Model 1. We also investigated the association of PA with depression risk across various strata defined by age, sex, BMI, race, economic level, marital status, and smoking status by using multivariable Model 2. All tests were considered significant at a *p*-value <0.05 (two-tailed). All analyses were completed using R statistical software (version 4.1.2; www.r-project.org).

## Results

In this study, we pooled a dataset that included 31,221 participants (15,377 males and 15,844 females) from NHANES 2007–2018 (Table 1 and Figure 1). The mean age was 48.22 (18.55) years. Of these participants, 13,821 (43.27%), 10,278 (32.92%), and 7,122 (22.81%) participants were young (aged 20–44 years), middle (aged 45–64 years), and older adults (aged ≥65 years), respectively. Overall, 2,837 (9.09%) participants were defined as having depressive symptoms in this population. According to the 2018 PA guidelines, 18,953 (60.71%) participants achieved the recommendation of total PA (≥150 min/week). In addition, 10,642 (34.09%), 4,550 (14.57%), and 10,570 (33.86%) participants achieved the recommendation of OPA, TPA, and LTPA, respectively. Detailed information is presented in Table 1.

**Table 1.** Characteristics of study population.

	All	Non-depression	Depression	*p*-value
Sample, *n* (%)	31,221	28,384 (90.91)	2,837 (9.09)	
Age, years, M (SD)	48.22 (18.55)	48.23 (18.69)	48.12 (17.07)	0.759
20–44	13,821 (43.27)	12,634 (44.51)	1,187 (41.84)	<0.001
45–64	10,278 (32.92)	9,129 (32.16)	1,149 (40.50)	
≥65	7,122 (22.81)	6,621 (23.33)	501 (17.66)	
Sex, *n* (%)				<0.001
Male	15,377 (49.25)	14,343 (50.53)	1,034 (36.45)	
Female	15,844 (50.75)	14,041 (49.47)	1,803 (63.55)	
Education, *n* (%)				<0.001
Less than college	13,838 (44.32)	12,220 (43.05)	1,618 (57.03)	
College or more	15,759 (50.48)	14,659 (51.65)	1,100 (38.77)	
BMI, kg/m^2^, *n* (%)				<0.001
Low to normal (<25)	9,129 (29.24)	8,429 (29.70)	700 (24.67)	
Overweight (25–30)	10,002 (32.04)	9,281 (32.70)	721 (25.41)	
Obese (≥30)	11,754 (37.65)	10,381 (36.57)	1,373 (48.40)	
Race/ethnicity, *n* (%)				<0.001
Mexican American	4,818 (15.43)	4,384 (15.45)	434 (15.30)	
Other Hispanic	3,256 (10.43)	2,864 (10.09)	392 (13.82)	
Non-Hispanic White	12,775 (40.92)	11,595 (40.85)	1,179 (41.56)	
Non-Hispanic Black	6,732 (21.56)	6,126 (21.58)	606 (21.36)	
Other race	3,640 (11.66)	3,414 (12.03)	226 (7.97)	
PIR, *n* (%)				<0.001
<1 (lowest income)	6,285 (20.13)	5,298 (18.67)	987 (34.79)	
1–1.99	7,664 (24.55)	6,839 (24.09)	825 (29.08)	
2–3.99	7,376 (23.63)	6,896 (24.30)	480 (16.92)	
≥4 (highest income)	7,069 (22.64)	6,802 (23.96)	267 (9.41)	
Marital status, *n* (%)				<0.001
Married	17,534 (56.16)	16,328 (57.53)	1,206 (42.51)	
Single	12,969 (38.66)	10,557 (37.19)	1,512 (53.30)	
Smoking, *n* (%)				<0.001
Nonsmoker	17,106 (54.79)	15,952 (56.20)	1,154 (40.68)	
Former smoker	7,366 (23.59)	6,736 (23.73)	630 (22.21)	
Current smoker	5,924 (18.97)	4,927 (17.36)	997 (35.14)	
Chronic disease, *n* (%)				
Hypertension	12,892 (41.29)	11,448 (40.33)	1,444 (50.90)	<0.001
Diabetes	5,318 (17.03)	4,626 (16.30)	692 (24.39)	<0.001
Arthritis	8,230 (27.84)	6,994 (24.64)	1,236 (43.57)	<0.001
Heart disease	2,513 (8.51)	2,095 (7.38)	418 (14.73)	<0.001
Stroke	1,113 (3.76)	898 (3.16)	215 (7.58)	<0.001
Pulmonary disease	5,986 (20.03)	5,038 (17.75)	948 (33.42)	<0.001
Cancer	2,925 (9.88)	2,614 (9.21)	311 (10.96)	0.005
Polypharmacy, *n* (%)	5,446 (17.45)	4,442 (15.65)	1,004 (35.39)	<0.001
Total PA: achieved, n (%)	18,953 (60.71)	17,670 (62.25)	1,383 (48.75)	<0.001
OPA: achieved, n (%)	10,642 (34.09)	9,741 (34.32)	901(31.76)	0.006
TPA: achieved, n (%)	4,550 (14.57)	4,146 (14.61)	404 (14.24)	0.617
LTPA: achieved, n (%)	10,570 (33.86)	10,047 (35.40)	523 (18.43)	<0.001

*Note: Unknown: education, 1,624 (5.20%); PIR, 2,827 (9.05%); marital status, 1,618 (5.18%); smoking, 825 (2.64%); hypertension, 1 (0.003%); arthritis, 1,657 (5.31%); heart disease, 1,690 (5.41%); stroke, 1,639 (5.25%); pulmonary disease, 1,340 (4.29%); cancer, 1,622 (5.20%); polypharmacy, 14 (0.04%).*

Abbreviations: LTPA, leisure-time PA; OPA, occupation-related PA; PA, physical activity; PIR, poverty income ratio; TPA, transportation-related PA.

**Figure 2.**
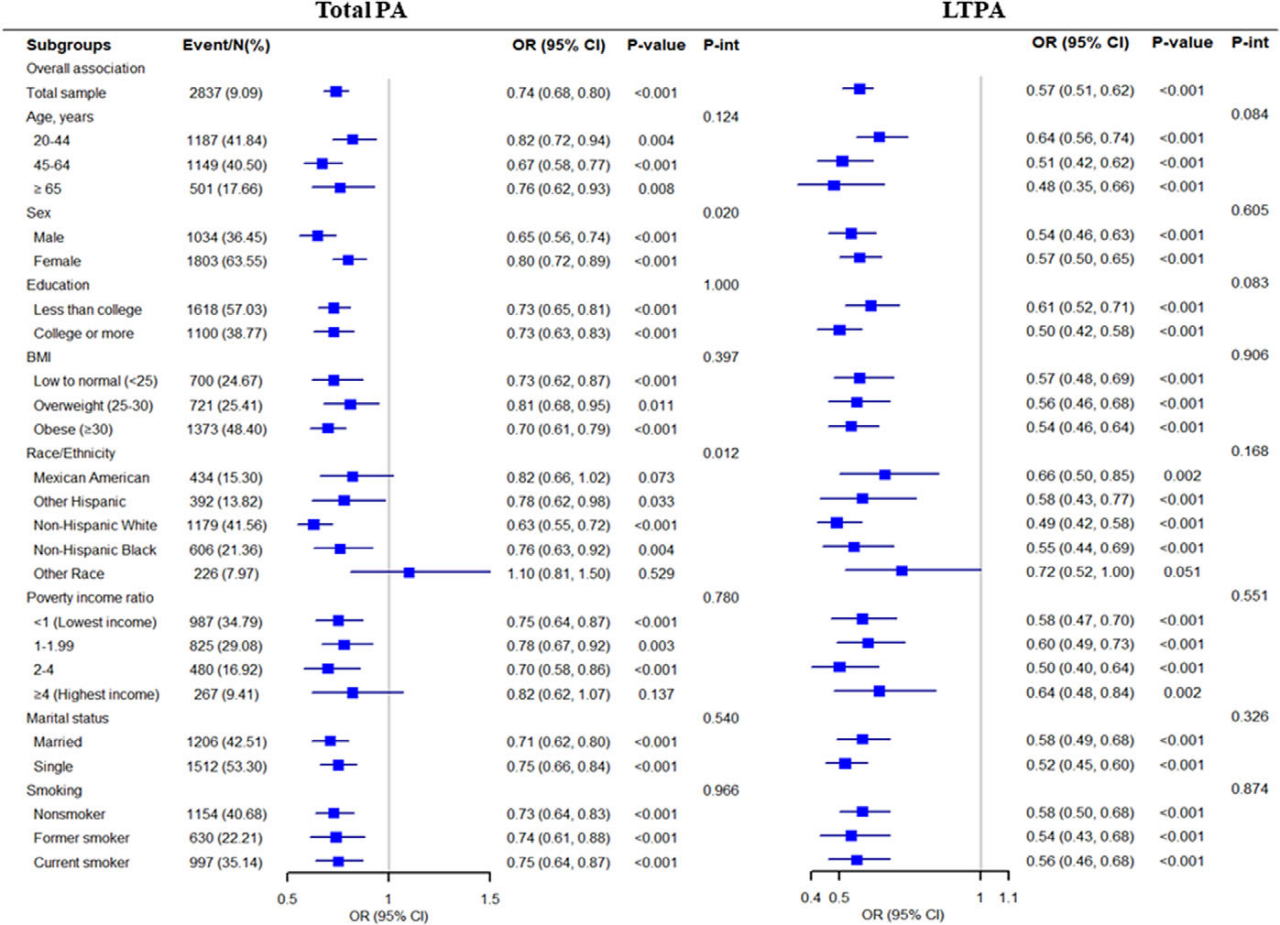
Association of total PA with LTPA based on meeting PA guidelines with risk of depression in subgroups. PA, physical activity; LTPA, leisure-time physical activity; OR, odds ratio; CI, confidence interval. All ORs were adjusted for age, sex, body mass index, race, education level, marital status, smoking status, poverty ratio, years of NHANES, stroke, diabetes, arthritis pulmonary disease, hypertension, heart disease, cancers, and polypharmacy. P_-int_ represents the heterogeneity between subgroups based on the meta-regression analysis.

Table 2 shows the results of multivariable logistic regression analyses between meeting PA guidelines for various domains of PA and risk of depressive symptoms. We found that total PA (odds ratio [OR] 0.61, 95% confidence interval [CI] 0.56–0.66) and LTPA (OR 0.42, 95% CI 0.38–0.46) that met the PA guidelines were inversely associated with the reduced risks of depressive symptoms in the age- and sex-adjusted model. After adjusting for demographic and metabolic confounders, those who met the PA guidelines for total PA had 26% lower odds of having depressive symptoms (OR 0.68, 95% CI 0.63–0.74), and those who met the PA guidelines for LTPA had 37% lower odds of having depressive symptoms (OR 0.53, 95% CI 0.47–0.58). After additional adjustment for chronic diseases, the associations of total PA (OR 0.74, 95% CI 0.68–0.80) and LTPA (OR 0.57, 95% CI 0.51–0.62) with depressive symptoms remained significant. However, OPA and TPA were not associated with depressive symptoms in the age- and sex-adjusted model or multivariable models in our analyses (Table 1 and Supplementary Tables S1 and S2). Furthermore, we conducted stratified analysis across various strata defined by age, sex, BMI, race, economic level, marital status, and smoking status using multivariable Model 2 (Figure 2). Excluding race, significant associations of total PA and LTPA with depressive symptoms were found in each stratum, and the interaction tests comparing the odds ratios across the strata were not significant (*p*-value >0.05), suggesting that LTPA was associated with a lower risk of depressive symptoms, regardless of age, sex, BMI, race, economic level, marital status, and smoking status.

**Table 2.** Multivariable OR for depression based on the meeting PA guideline.

	Event/*n* (%)	Age- and sex-adjusted model		Multivariable model 1		Multivariable model 2	
		OR (95% CI)	*p*-value	OR (95% CI)	*p*-value	OR (95% CI)	*p*-value
Total PA							
No	1,454/12,268 (11.85)	1 (Ref)	1 (Ref)	1 (Ref)	1 (Ref)	1 (Ref)	1 (Ref)
Yes	1,383/18,953 (7.30)	0.61 (0.56, 0.66)	<0.001	0.68 (0.63, 0.74)	<0.001	0.74 (0.68, 0.80)	<0.001
Occupation-related PA							
No	1,936/20,579 (9.41)	1 (Ref)	1 (Ref)	1 (Ref)	1 (Ref)	1 (Ref)	1 (Ref)
Yes	901/10,642 (8.47)	0.96 (0.89, 1.05)	0.390	0.91 (0.84, 1.00)	0.042	0.95 (0.87, 1.03)	0.216
Transportation-related PA							
No	2,433/26,671 (9.12)	1 (Ref)	1 (Ref)	1 (Ref)	1 (Ref)	1 (Ref)	1 (Ref)
Yes	404/4,550 (8.88)	1.03 (0.92, 1.15)	0.659	0.95 (0.84, 1.06)	0.353	1.02 (0.91, 1.15)	0.682
Leisure-time PA							
No	2,314/20,651 (11.21)	1 (Ref)	1 (Ref)	1 (Ref)	1 (Ref)	1 (Ref)	1 (Ref)
Yes	523/10,570 (4.95)	0.42 (0.38, 0.46)	<0.001	0.53 (0.47, 0.58)	<0.001	0.57 (0.51, 0.62)	<0.001

*Note: Multivariable model 1 was adjusted for age, sex, body mass index, race, education level, marital status, smoking status, poverty ratio, and years of NHANES; Multivariable model 2 was adjusted for stroke, diabetes, arthritis pulmonary disease, hypertension, heart disease, cancers, and polypharmacy in addition to model 1.*

Abbreviations: CI, confidence interval; OR, odds ratio; PA, physical activity.

**Figure 3.**
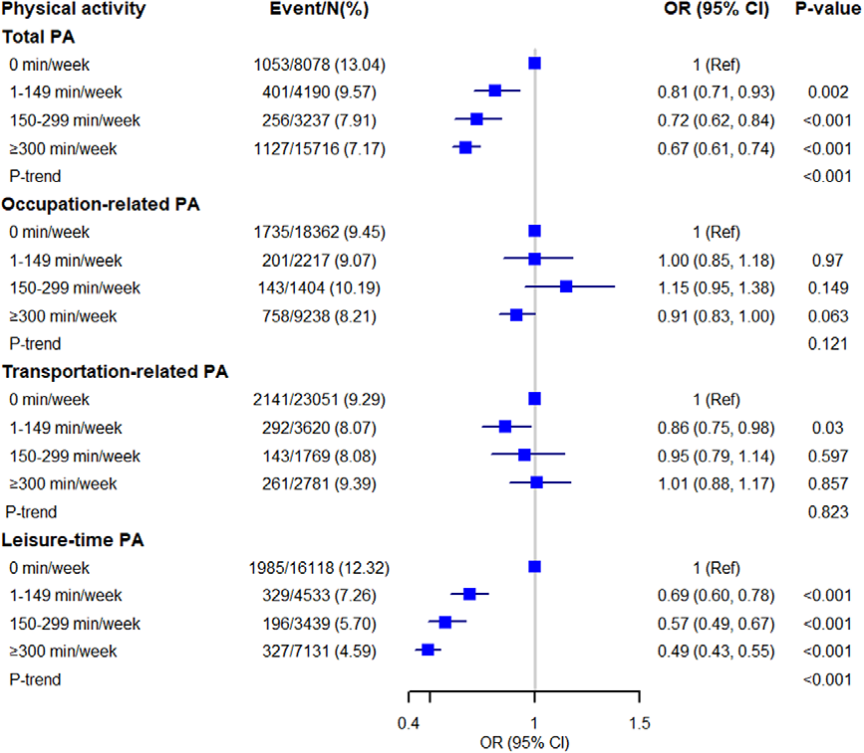
Multivariable OR for depression based on the amount of PA. PA, physical activity; OR, odds ratio; CI, confidence interval. All ORs were adjusted for age, sex, body mass index, race, education level, marital status, smoking status, poverty ratio, years of NHANES, stroke, diabetes, arthritis pulmonary disease, hypertension, heart disease, cancers, and polypharmacy.

Additionally, we categorized the amount of PA into four groups (0, 1–149, 150–299, and ≥300 min/week) to assess the potential dose–response relationships between various PA domains and depressive symptoms and to evaluate the additional benefits of PA beyond or below the PA guidelines (Figure 3). Similar inverse associations across LTPA categories and depressive symptoms were also found. After adjusting for confounders, participants who performed <1 time (1–149 min/week), 1–2 times (150–299 min/week), or over two times (≥300 min/week) the recommended level of PA guidelines had 31% (OR 0.69, 95% CI, 0.60–0.78), 43% (OR 0.57, 95% CI, 0.49–0.67), and 51% (OR 0.49, 95% CI, 0.43–0.55) lower risks for depressive symptoms, respectively. These findings suggested that LTPA was associated with lower risks of depressive symptoms regardless of level (1–149, 150–299, and ≥300 min/week). However, no significant associations of TPA and OPA with depressive symptoms were found (*p*-value >0.05).

## Discussion

In this nationally representative cross-sectional survey of NHANES, we found that meeting the PA guideline for LTPA was associated with a reduced risk of depressive symptoms, regardless of age, gender, BMI, race, economic level, marital status, and smoking status. Interestingly, LTPA, but not OPA or TPA, was associated with a lower risk of depressive symptoms at any amount, suggesting that significant mental health would benefit from PA, even at levels below the PA recommendations. However, no significant associations of TPA and OPA with depressive symptoms were found.

At present, most studies have focused on the association of total PA or LTPA with depressive symptoms, and few studies have investigated whether the same health-enhancing relationships are observed across different PA domains in the general population [12–14]. It remains unclear whether all PA domains (e.g., OPA, TPA, and LTPA) have equivalent beneficial relationships on depressive symptoms. Previous observational studies have demonstrated a significant association of total PA with depressive symptoms, suggesting a potentially modifiable lifestyle for prevention [12, 13]. In a recent meta-analysis of 15 prospective cohort studies including 191,130 participants and 2,110,588 person-years, Pearce et al. [12] found an inverse curvilinear dose–response association between total PA and depressive symptoms. Compared to adults not reporting any activity, those engaged in half the recommended volume of PA (4.4 marginal metabolic equivalent task hours per week [mMET-h/wk]) had an 18% (OR 0.82, 95% CI 0.77–0.87) lower risk of depressive symptoms. Adults engaged in the recommended volume of 8.8 mMET-h/wk had a 25% (OR 0.75, 95% CI 0.68–0.82) lower risk of depressive symptoms, and adults engaged in twice the recommended volume of 17.5 mMET-h/wk had a 28% (OR 0.72, 95% CI 0.64–0.81) lower risk of depressive symptoms [12]. Their findings were consistent with our results that total PA was associated with lower risks of depressive symptoms, regardless of amount (1–149, 150–299, and ≥300 min/week) of PA. In parallel, Mendelian randomization studies also validated a potential protective role of PA on depressive symptoms [15, 16]. Choi et al. [15] conducted a two-sample Mendelian randomization study, which included two PA phenotypes, self-reported (*n* = 377,234) and objective accelerometer-based (*n* = 91,084), to assess bidirectional relationships between PA and major depressive disorder (*n* = 143,265) among adults. They found a protective relationship between objectively assessed PA (OR, 0.74 per 1-SD increase in mean acceleration, 95% CI 0.59–0.92) but not self-reported PA (OR 1.28 per 1-SD increase in metabolic-equivalent minutes of reported moderate-to-vigorous activity; 95% CI 0.57–3.37) and the risk for major depressive disorder [15]. However, there was no significant association of major depressive disorder with self-reported or objectively assessed PA (*p*-value >0.05) [15]. Their findings supported the hypothesis that enhancing PA may be an effective prevention strategy for depression.

Additionally, a recent meta-analysis of 111 prospective cohort studies, which included over 3 million adults sampled from 11 nations on five continents, reported that customary and increasing levels of LTPA (OR 0.80, 95% CI 0.77–0.83) are inversely associated with incident depression among adults, regardless of global region, gender, age, or follow-up period [13]. Similarly, we found that meeting the PA guideline for LTPA was associated with a reduced risk of depressive symptoms, regardless of age, gender, BMI, race, economic level, marital status, and smoking status. However, in our study, we analyzed the relationship between the total amount but not the detailed types of LTPA and depression. Choi et al. [32] conducted a longitudinal biobank cohort study of 7,968 individuals of European ancestry and assessed eight different types of LTPA: walking/hiking (including commuting for work), jogging, running, biking, racquet sports, swimming, high‐intensity exercise (e.g., dance and aerobics), and low‐intensity exercise (e.g., yoga and stretching). They found that running (OR 0.87 per 1-SD increase, 95% CI 0.78–0.96, *p*-value = 0.013) and walking (OR = 0.89 per 1-SD increase, 95% CI 0.80–0.97, *p*-value = 0.013) showed nominal associations with incident depression, even after adjusting for polygenic risk. They did not find significant associations for jogging, biking, racquet sports, or swimming [32]. A Mendelian randomization study, which consisted of 123,794 adults of white British ancestry who enrolled in the UK Biobank, found that exercises (e.g., swimming, cycling) (beta = 0.90, *p*-value = 0.033), but not walking for pleasure (beta = 1.01, *p*-value = 0.765), were associated with depression. They also found that depression was associated with walking for pleasure (beta = 1.06, *p*-value = 0.036) rather than exercise (e.g., swimming and cycling) (beta = 0.98, *p*-value = 0.452). Due to the self-reported PA reflecting average levels in the past year, the true levels of PA may fluctuate over the course of a year in their study. The significant association of walking with depression may be attributed to reverse causality or confounding factors. In summary, evidence from both observational and Mendelian randomization studies has provided evidence that LTPA may be an effective prevention strategy for depression.

Unfortunately, neither OPA nor TPA was associated with depressive symptoms in our analyses. Few studies have investigated whether the same health-enhancing relationships are observed across different PA domains [24, 33–35]. A U-shaped relationship between total PA and incident depressive symptoms was found in the Kangbuk Samsung Health Study, which included 119,069 Korean adults in the age group of 18–64 years [34]. Chen et al. [36] included 2,727 participants aged ≥65 years participating in the 2005 Taiwan National Health Interview Survey and found that LTPA (0 kcal/week: OR 3.72, 95% CI 2.28–6.06; 1–999 kcal/week: OR 2.06, 95% CI 1.25–3.39), but not non-LTPA (e.g., farm work, heavy lifting, fishing work, and household chores), was significantly associated with depressive symptoms compared with 2,000 kcal/week. A cross-sectional study conducted in the Brazilian National Health Survey (*n* = 60,202; ≥18 years) also explored associations of PA domains with risks of depressive symptoms [35]. They found that TPA was also associated with lower depressive symptoms among older adults (OR 0.70, 95% CI 0.53–0.94) but not young or middle-aged adults (OPA) (OR 1.62, 95% CI 1.38–1.91) [35]. We suspect that the reasons for the inconsistent results may result from disparities in ethnicity and education. Choi et al. [16] also found that there were no causal associations of walking for pleasure (beta 1.02, 95% CI 0.92–1.12), transport by walking (beta 0.98, 95% CI 0.87–1.11), or frequency of walking (beta 1.02, 95% CI 0.85–1.123) with depression in a Mendelian randomization study. In summary, the association of OPA and TPA with depressive symptoms is still controversial and should be validated in further studies.

Our study has several strengths. We assessed the association with depressive symptoms for total PA but also for PA domains (OPA, TPA, and LTPA). Because it remains unclear whether all PA domains have equivalent beneficial relationships at present, we responded to this knowledge gap. We found that LTPA, rather than OPA or TPA, was associated with depressive symptoms in adults across a wide age spectrum. Our results were based on the NHANES, a nationally representative cross-sectional survey of civilian, noninstitutionalized persons living in the USA. Therefore, our findings can be generalized to the U.S. population, regardless of age, gender, BMI, race, economic level, marital status, and smoking status. However, there were several limitations in our study. First, the NHANES data are cross-sectional, and we did not examine causal relationships between PA domains and depressive symptoms due to reverse causality and unknown confounding factors. In further studies, prospective and Mendelian randomization studies are needed to assess the potential roles of PA domains on depressive symptoms to validate our findings. Second, the PA domains were assessed by self-report questionnaires rather than objective measurements, and the number of PA domains was assessed for a typical week at a single time. We could not capture the stable levels or trajectories of PA domains. Further studies should collect consecutive data on PA domains by objective measurements to investigate the association of PA with depression. Finally, depressive symptoms were assessed by the PHQ-9, which is a self-report questionnaire. The accurate assessment of depression by clinical diagnosis is needed in the future.

## Conclusion

In this nationally representative cross-sectional survey of NHANES 2007–2018, we showed different relationships of PA domains on depressive symptoms. LTPA, but not OPA or TPA, at any amount was associated with a lower risk of depressive symptoms. The type (LTPA, TPA, and OPA), intensity (vigorous, moderate, or combination) and amount of PA must be assessed independently, and recommendations should be individualized. In summary, our study suggests that increasing LTPA, even at levels below the PA recommendations, should be suggested as a potential lifestyle modification to prevent or treat depressive symptoms. Longitudinal studies and objective measurements of PA are needed to validate our findings in the future.

## Data Availability

The datasets analyzed in the study are publicly available at https://www.cdc.gov/nchs/nhanes/index.htm. The datasets generated and analyzed during the current study are available from the corresponding author on reasonable request.

## Supplementary material

For supplementary material accompanying this paper visit https://doi.org/10.1192/j.eurpsy.2022.2350. click here to view supplementary material
