# Supplementary material for: Association of domain-specific physical activity with depressive symptoms: A population-based study
Source: Eur Psychiatry. 2022 Dec 12;66(1):e5. doi: 10.1192/j.eurpsy.2022.2350 (PMC9879900; doi:10.1192/j.eurpsy.2022.2350)
Supplement: Supplementary file 1 [file S0924933822023501sup001.docx]

**Supplemental Table 1 The association between the OPA based on the Meeting Physical Activity Guideline and risk of depression stratified by subgroups**

| Subgroup | OR (95% CI) | P value | P-interaction |
| --- | --- | --- | --- |
| Age, years |  |  | 0.553 |
| 20-44 | 0.98 (0.86, 1.12) | 0.778 |  |
| 45-64 | 0.88 (0.76, 1.02) | 0.096 |  |
| ≥ 65 | 0.96 (0.76, 1.21) | 0.741 |  |
| Sex, n (%) |  |  | 0.026 |
| Male | 0.82 (0.72, 0.95) | 0.006 |  |
| Female | 1.06 (0.95, 1.19) | 0.305 |  |
| Education, n (%) |  |  | 0.008 |
| Less than college | 0.80 (0.71, 0.91) | 5.29E-4 |  |
| College or more | 1.03 (0.89, 1.18) | 0.706 |  |
| BMI, kg/m^2^, n (%) |  |  | 0.109 |
| Low to normal (<25) | 0.97 (0.82, 1.15) | 0.715 |  |
| Overweight (25-30) | 1.07 (0.90, 1.27) | 0.452 |  |
| Obese (≥30) | 0.85 (0.74, 0.97) | 0.015 |  |
| Race/Ethnicity, n (%) |  |  | 0.372 |
| Mexican American | 1.06 (0.84, 1.34) | 0.598 |  |
| Other Hispanic | 0.96 (0.74, 1.23) | 0.755 |  |
| Non-Hispanic White | 0.85 (0.74, 0.98) | 0.024 |  |
| Non-Hispanic Black | 0.98 (0.80, 1.18) | 0.810 |  |
| Other Race | 1.11 (0.81, 1.52) | 0.501 |  |
| Poverty income ratio, n (%) |  |  | 0.362 |
| <1 (Lowest income) | 0.93 (0.79, 1.09) | 0.372 |  |
| 1-1.99 | 1.01 (0.86, 1.19) | 0.902 |  |
| 2-4 | 0.86 (0.70, 1.06) | 0.163 |  |
| ≥4 (Highest income) | 1.14 (0.87, 1.48) | 0.351 |  |
| Marital status, n (%) |  |  | 0.288 |
| Married or living with partner | 0.86 (0.75, 0.98) | 0.024 |  |
| Single | 0.95 (0.84, 1.08) | 0.467 |  |
| Smoking, n (%) |  |  | 0.631 |
| Nonsmoker | 0.92 (0.80, 1.06) | 0.271 |  |
| Former smoker | 0.98 (0.81, 1.18) | 0.819 |  |
| Current smoker | 0.87 (0.74, 1.01) | 0.064 |  |

PA, physical activity; OR, odds ratio; CI, confidence interval; All models were adjusted for age, sex, body mass index, race, education level, marital status, smoking status, poverty ratio, years of NHANES, stroke, diabetes, arthritis pulmonary disease, hypertension, heart disease, cancers and polypharmacy.

**Supplemental Table 2 The association between the TPA based on the Meeting Physical Activity Guideline and risk of depression stratified by subgroups**

| Subgroup | OR (95% CI) | P value | P-interaction |
| --- | --- | --- | --- |
| Age, years |  |  | 0.658 |
| 20-44 | 1.07 (0.91, 1.26) | 0.393 |  |
| 45-64 | 0.98 (0.80, 1.19) | 0.831 |  |
| ≥ 65 | 1.16 (0.81, 1.61) | 0.406 |  |
| Sex, n (%) |  |  | 0.124 |
| Male | 1.12 (0.94, 1.33) | 0.180 |  |
| Female | 0.93 (0.79, 1.09) | 0.381 |  |
| Education, n (%) |  |  | 0.702 |
| Less than college | 1.05 (0.90, 1.23) | 0.539 |  |
| College or more | 1.00 (0.82, 1.21) | 0.987 |  |
| BMI, kg/m^2^, n (%) |  |  | 0.399 |
| Low to normal (<25) | 1.14 (0.93, 1.39) | 0.194 |  |
| Overweight (25-30) | 0.98 (0.78, 1.23) | 0.884 |  |
| Obese (≥30) | 0.95 (0.78, 1.14) | 0.567 |  |
| Race/Ethnicity, n (%) |  |  | 0.065 |
| Mexican American | 0.83 (0.61, 1.12) | 0.243 |  |
| Other Hispanic | 1.01 (0.74, 1.34) | 0.969 |  |
| Non-Hispanic White | 1.01 (0.83, 1.23) | 0.894 |  |
| Non-Hispanic Black | 0.95 (0.74, 1.22) | 0.704 |  |
| Other Race | 1.63 (1.14, 2.31) | 0.007 |  |
| Poverty income ratio, n (%) |  |  | 0.443 |
| <1 (Lowest income) | 0.93 (0.77, 1.13) | 0.477 |  |
| 1-1.99 | 0.96 (0.76, 1.20) | 0.745 |  |
| 2-4 | 1.13 (0.83, 1.50) | 0.422 |  |
| ≥4 (Highest income) | 1.25 (0.85, 1.80) | 0.240 |  |
| Marital status, n (%) |  |  | 0.880 |
| Married or living with partner | 1.04 (0.85, 1.26) | 0.685 |  |
| Single | 1.02 (0.87, 1.19) | 0.829 |  |
| Smoking, n (%) |  |  | 0.262 |
| Nonsmoker | 1.05 (0.88, 1.26) | 0.571 |  |
| Former smoker | 0.83 (0.61, 1.10) | 0.210 |  |
| Current smoker | 1.11 (0.91, 1.34) | 0.315 |  |

PA, physical activity; OR, odds ratio; CI, confidence interval; All models were adjusted for age, sex, body mass index, race, education level, marital status, smoking status, poverty ratio, years of NHANES, stroke, diabetes, arthritis pulmonary disease, hypertension, heart disease, cancers and polypharmacy.
